# Midwives' Physiological Approach at the Third Stage of Labour: A Scoping Review

**DOI:** 10.1111/birt.70048

**Published:** 2026-01-06

**Authors:** Elena Tarlazzi, Virginia Berini, Lorenzo Brevi, Rosalba Ferrandino, Mara Tormen, Dila Parma, Rosaria Cappadona, Simona Fumagalli, Antonella Nespoli, Giuliana Simonazzi

**Affiliations:** ^1^ Department of Medical and Surgical Sciences (DIMEC) University of Bologna Bologna Italy; ^2^ AUSL Romagna, Direzione delle Professioni sanitarie_Ambito Rimini Rimini Italy; ^3^ Policlinico di Sant'Orsola, IRCCS Azienda Ospedaliero‐Universitaria di Bologna Bologna Italy; ^4^ Ausl Romagna_Obstetric Unit_Ambito Rimini Rimini Italy; ^5^ Capobianco Group_Centro per la Diagnosi e Terapia Della sterilità di Coppia Apricena, Puglia Italy; ^6^ Department of Medical Sciences Institute of Obstetrics and Gynecology, University of Ferrara Ferrara Italy; ^7^ School of Medicine and Surgery University of Milano‐Bicocca Monza Italy; ^8^ Department of Obstetrics Foundation IRCCS San Gerardo dei Tintori Monza Italy

**Keywords:** midwifery, natural childbirth, parturition, professional practice, third labour stage

## Abstract

**Introduction:**

The third stage of labour occurs between the birth of the foetus and the expulsion of the placenta. A major complication during this stage is postpartum hemorrhage, which poses a significant concern for maternal health. To mitigate this risk, active management strategies have emerged, raising concerns about the safety of physiological management following a physiological birth. Midwives play a vital role in ensuring a safe third stage of labour by minimizing medical intervention and preventing postpartum hemorrhage. This scoping review aims to map the evidence on midwifery practices that support a physiological third stage of labour.

**Methods:**

We conducted a scoping review using the Joanna Briggs Institute methodology. We retrieved articles from PUBMED, PsycINFO (via EBSCO), CINAHL (via EBSCO), LILACS, SCOPUS, ClinicalTrials.gov, Open Science Framework, ProQuest Dissertations and Theses, Cochrane Library, and JBI.

**Results:**

The search yielded 2190 articles, with 1779 remaining after duplicates were removed. Screening identified 80 articles for review; 63 were excluded, resulting in 17 articles defining key findings. Two articles contributed to a theoretical framework for a physiological approach. Ten articles further discussed how midwives provide care during this stage to maintain normalcy. Three articles debated the safety of expectant management for low‐risk women, while three studies also suggested new risk factors for postpartum hemorrhage.

**Conclusion:**

Midwifery care during the physiological third stage of labour must balance safety and trust in the woman's body. More research is needed to assess expectant management in home births and midwifery‐led units, with a focus on individual needs and the long‐term impacts of a physiological approach.

## Graphical abstract

Midwives play a crucial role in supporting a physiological third stage of labour by balancing trust in the woman's body with safety measures to prevent postpartum haemorrhage. This scoping review identifies key midwifery practices that promote natural placenta and highlights the importance of undisturbed, woman‐centred care.

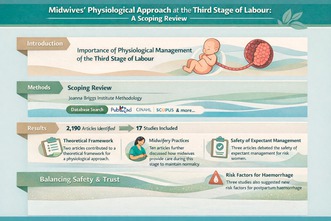

## 1 Introduction

Midwifery care has a key role in optimizing the woman's reproductive psychophysiology during childbirth. As outlined by Renfrew et al. [1], midwifery care effectively promotes the normal childbirth process and helps prevent complications. Experiencing a physiological labour and birth may contribute to positive outcomes for the mother, the baby, and the whole family [2]. According to the WHO guidelines, a positive birth experience comes through respectful care, continuity of care, effective communication, and reduction of medical intervention.

In this context, postpartum hemorrhage (PPH) emerges as a significant global health concern, affecting millions of women annually and accounting for over 20% of maternal deaths worldwide [3]. Despite its prevalence, most PPH‐related fatalities are preventable, particularly in high‐income countries where interventions have nearly eradicated these deaths. However, the widespread use of interventions during placental birth seems at odds with the growing focus on protecting physiological birth processes.

Midwives play a key role in keeping this stage of labour physiologic and safe [4]. While much research has focused on the active management of the third stage, midwives can significantly contribute to minimizing medical intervention and preventing postpartum hemorrhage by supporting natural physiological processes, as described in the hormonal and emotional pathways by Dixon et al. [5, 6]. Additionally, many women consider a natural birth of the placenta an essential element of a physiological birth [7].

According to the World Health Organization (WHO) guidelines, expectant management includes avoiding the routine use of uterotonic drugs, delaying cord clamping until pulsation stops, and relying on maternal effort for placenta delivery, World Health Organization [8]. Recent updates to a Cochrane systematic review [9] comparing active and expectant management of the third stage of labour have provided new insights. In high‐income settings, active management appears to reduce mean maternal blood loss and the need for therapeutic uterotonics. However, it may also introduce negative effects, such as, discomfort, bleeding complications, and postnatal hypertension. The review's conclusions differ from earlier versions (Begley et al. [9)], which declared that active treatment was “superior” to expectant management. Moreover, new insights on care during the third stage emerged from the review's conclusions. First, there is no clear definition of what constitutes expectant management; therefore, practice varies widely. Second, both women at low and high risk of postpartum hemorrhage have been involved. Finally, not all midwives were equally skilled in expectant management of the third stage.

Given the complexity of managing the third stage of labour, our research group has conducted a scoping review to map available evidence on midwifery practices that support a physiological third stage of labour.

## 2 Materials and Methods

### 2.1 Study Design

Using a scoping review process, it was possible to map important concepts and features of the phenomena from a wide range of sources (Pollock et al. [10]. The review used the Preferred Reporting Items for Systematic Reviews and Meta‐Analyses extension for Scoping Reviews (PRISMA‐ScR; Tricco et al. [11]) and the Joanna Briggs Institute (JBI) scoping review methodology, which was improved by Peters et al. [12]. The review included the following five key phases: (1) identifying the research question, (2) identifying relevant studies, (3) study selection, (4) charting the data, and (5) collating, summarizing, and reporting the results. The optional ‘consultation exercise’ of the framework was not conducted. A detailed review protocol was published on Open Science Framework (doi:10.17605/OSF.IO/XHD35).

### 2.2 Review Question(s)

This scoping review aims to map the evidence on midwifery practices that support a physiological third stage of labour.

The sub questions defining the objectives of this study are:

1. What defines a normal third stage of labour?
2. How do midwives provide care during the third stage of labour to keep it normal?
3. What evidence supports a physiological approach to managing the third stage of labour?

### 2.3 Information Sources and Search Strategy

The search strategy was developed by the authors and a librarian at the University of Bologna. The research was conducted in the following databases: PUBMED, Psycinfo (via EBSCO), CINAHL (via EBSCO), LILACS, SCOPUS, ClinicalTrial.gov, Open Science Framework, ProQuest Dissertation and Thesis, Cochrane Library, and JBI. Midwifery‐relevant journals have also been retrieved, such as, Midwifery, Women and Birth, Birth, British Journal of Midwifery, BMC Pregnancy and Childbirth, as well as conference proceedings on birth physiology or midwifery care. Moreover, the reference lists of relevant studies identified throughout the search process were scanned.

The PCC (Population; Concept; Context) framework was utilized to facilitate the establishment of the inclusion and exclusion criteria as well as the search terms (see Table 1).

**TABLE 1:** Search terms and inclusion/exclusion criteria mapped to the PEO framework.

Criteria	Inclusion criteria	Exclusion criteria	Search terms
Study population	Midwives, obstetric nurses, nurse midwives and skilled birth attendants	Students midwives, obstetrics, Physicians, Nurse	((“midw*”[Title/Abstract] OR “nurse midwi*”[Title/Abstract] OR “obstetric nurs*”[Title/Abstract] OR (“nurse midwives”[MeSH Terms] OR “midwifery”[MeSH Terms] OR “obstetric nursing”[MeSH Terms])))
Exposure to context	Physiological care during third stage of labour is defined as the variety of emotional and physical actions midwives do	Studies on PPH management and OR PPH prevention will be excluded, as well as studies on women's experience	((holistic care) OR (physiological care)) OR ((“skill*”[Title/Abstract] OR “practice*”[Title/Abstract] OR “method*”[Title/Abstract] OR “technique*”[Title/Abstract] OR “role”[Title/Abstract] OR “care”[Title/Abstract] OR “help”[Title/Abstract] OR “support*”[Title/Abstract] OR “supportive care”[Title/Abstract])) OR (“obstetric nursing/methods”[MeSH Terms] OR “midwifery/methods”[MeSH Terms]) AND ((“Placenta”[Mesh]) OR “Placenta/physiology”[Mesh]) OR ((third stage labor) OR (third stage labour)) OR (placent*[Text Word]) OR (“Labor Stage, Third”[Mesh])
Date	No data restriction		
Study type	All research methodology, qualitative reviews, quantitative reviews, literature reviews and unpublished literature will be included, as well as guidelines, policy documents, position statements	Personal opinion	
Language	No language restriction		

The PCC framework as applied to the scoping review included.

### 2.4 Population

This scoping review considered all studies that focus on midwives caring for women during the third stage of labour. All over the world, midwifery education differs widely; therefore, we decided to include studies including midwives, obstetric nurses, nurse midwives, and skilled birth attendants.

### 2.5 Concept

Physiological care is defined as the variety of emotional and physical actions midwives do when “being with the woman, in a watchful attendance” [13].

### 2.6 Context

Studies were conducted in all clinical settings (home, hospital, midwifery Unit) and irrespective of the countries.

The search strategy combined indexed terms (MeSH) and free text terms for the concepts in the study. All research methodology, qualitative reviews, quantitative reviews, literature reviews, and unpublished literature have been included. Studies were included if they reported the description of midwifery care or the midwifery management of a normal third stage of labour, as well as the variety of emotional and physical actions midwives undertake when “being with the woman” [13] during the third stage of labour in any clinical setting. Studies focusing only on PPH management and PPH prevention have been excluded, as have studies on women's experiences of care during the third stage and those conducted in low‐income countries. All over the world, midwifery education differs widely; therefore, researchers decided to include studies involving midwives, obstetric nurses, and nurse midwives. No data or language restrictions have been applied.

### 2.7 Study/Sources of Evidence Selection

**FIGURE 1:**
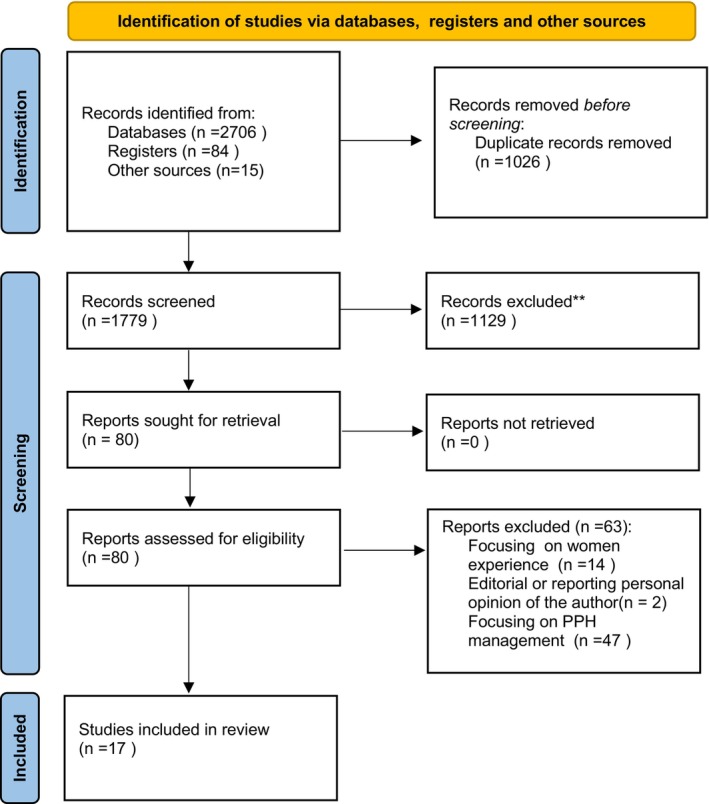
The PRISMA flow diagram. PRISMA 2020 flow diagram for new systematic reviews which included searches of databases and registers only. *Source:* Page MJ, et al. BMJ 2021;372:n71. doi: 10.1136/bmj.n71. This work is licensed under CC BY 4.0. and retrievable at https://www.prisma‐statement.org/prisma‐2020‐flow‐diagram. [Colour figure can be viewed at wileyonlinelibrary.com]

The first author (ET) ran the database searches, with potentially relevant articles downloaded into Rayyan (a web‐based tool that supports collaborative systematic reviews—https://www.rayyan.ai/). After duplication was removed, two authors (VB and LB) carried out title and abstract screening to weed out unrelated studies. Any disagreements were settled by discussing with a third author (ET) until an agreement was achieved. After that, two author couples (VB and LB or ET and RF) completed the entire text screening; once more, a third author (MT) was brought in to help with perspective and conflict resolution. Figure 1 provides the PRISMA flow chart of the literature search process.

The initial database searches were undertaken in July 2023. Alerts were set up with the databases to ensure notification of recent publications related to the search terms. The literature search and screening process were conducted between July and December 2023.

### 2.8 Data Extraction

Two authors (ET, RF) independently extracted data from full‐text papers using a data extraction tool template. The data extracted included publication details such as, authors, country, year of publication, methods, population, concept, context, and key findings and/or recommendations. For a chart of the listed studies, see Table 2.

**TABLE 2:** Results of review.

Author, Year, Country (if applicable)	Aim of the study	Design, methods	Expectant management (EMTSL)	Active management (AMTSL)	Participants	Key fundings
Featherstone, 1999, UK	To find out to what extent midwives understand the conduct of a physiological third stage	Exploratory study, with a multiple choice questionnaire	Mother's informed choice. No cord traction. Await spontaneous separation and delivery of placenta. Give (intramuscular syntometrine if placenta remains undelivered after 40 min)	Nd	78 hospital midwives	The study divided management strategies into nine categories based on data and literature review. The categories included giving oxytocic agents, applying controlled cord traction, allowing placenta and membranes to be delivered by maternal effort and gravity, cutting and clamping the cord, feeling for the delivery by maternal effort and gravity, clamping and cutting the cord once pulsation ceases, releasing the clamp on the maternal end of the cord, not feeling for the delivery by maternal effort and gravity, and leaving the cord intact
Harris, 2005	To explore the concept of practice variation in third stage management among midwives, focusing on what midwives do and why they do it	Grounded Theory with multiple data collection	Management without uterotonics (though these were often made ready just in case they were needed)	A package of care involving the administration of an uterotonic drug	Fifty one midwives employed in two NHS midwives working in a wide range of environments, of differing levels of expertise, and with differing values and beliefs	Despite the naming of two categories of practice (expectant vs. active management), midwives described multiple ways of managing the third stage of labour. The complexity of third stage care was revealed by the identification of 22 aspects or parts to care. A theory of contingent decision making for the third stage of labour was revealed which explained how midwives adopted different forms of care through a complex decision making process which was contingent on the learning opportunities midwives were exposed to, the context in which practice decisions were made and the philosophical underpinnings of midwifery care
Tan, 2008; British Columbia	To compare practices of three maternity care provider disciplines in management of third‐stage labor and the justifications for their approach	Cross‐sectional survey with an online multiple choices questionnaire	NA	NA	287 out of 497 Obstetricians and family physicians and midwives	Midwives are less likely to perform early cord clamping and cutting, use prophylactic oxytocin for third‐stage labour management, and perform uterine massage after placenta delivery compared to obstetricians and family physicians. Midwives wait for spontaneous separation and prefer the expectant approach.
Fahy, 2010	The study examines existing RCT evidence comparing physiological and active management of third stage labor care for low‐risk women to evaluate its relevance and validity	Literature review	Different description of what constitutes EMTSL	Different definition of what constitute AMTSL	n.a.	Actual research doesn't provide evidence about what constitutes and the effectiveness of physiological third stage care
Dixon, 2009, New Zealand	To describe, analyze, and compare the outcomes of expectant or active management for the third stage of labour following a normal physiological birth	Prospective cohort study	To promote a woman's body's endogenous oxytocin production through skin‐to‐skin contact, warmth, and calmness, aiming to enhance physiological processes for holistic health support, with only necessary treatment intervention	Exogenous oxytocin administration	33,752 women in the NZCOM	The research involved 16,238 women who received physiological management (48.1%) and 17,514 (51.9%) who received active management for placental delivery. Women delivering at home or in primary birthing units were more likely to have a physiological third stage Most women lost less than 500 mL of blood, with the majority receiving physiological support (96.3%). 11.3% of the physiological management group required more time to complete the third step
Hastie, 2009	To describe midwifery led care in the third stage of labour, based on the midwifery Guardianship theory	Theory description	n.a.	n.a.	n.a.	Placental birth is a crucial process that requires a safe environment for both mother and infant. The ideal psycho‐physiological state for birth is a calm and connection state, where the parasympathetic system is dominant. Midwifery guardians should promote maternal–infant love ensuring a natural physiological placental birth
Jangsten, 2010; Sweden	To explore Swedish midwives' experiences of management of the third stage of labour	Qualitative design with focus group discussions	Prophylactic intravenous injection of 10 units of Oxytocin as soon as the infant is born to all women, but do not perform the entire active management third stage labour procedure	Active management third stage labour	32 midwives with more than 6 years' experience in labour room	The analysis generated three categories: ‘bring the process under control’, ‘protect normality and women's birthing experiences’ and ‘maintain midwives’ autonomy’
Fahy et al., 2010 Australia	To compare the PPH rates of a tertiary referral maternity unit, and a midwifery‐led, freestanding birthing	Retrospective cohort study	Holistic psychophysiological third stage care	I.M. Syntocinon should be given within 1 min of the birth of the baby; controlled cord traction is to be used followed by fundal massage after the placenta is born	The total number of women who were at low risk of PPH was 3436 comprising 3075 at the tertiary unit and 361 at the midwifery‐led unit, between July 2005 and August 2008	The intention‐to‐treat analysis shows a PPH rate of 11.2% for active management of the third stage of labour at the tertiary unit compared with 2.8% for holistic psychophysiological care at the midwife‐led unit OR = 4.4, 95% CI [2.3, 8.4]
Dixon, 2011	To establish the clinical effectiveness of physiological third‐stage care following a physiological labor and birth	Systematic review			2 RCT 2 Observational study included, involving 35,455 healthy women	The study found minimal differences in blood loss volumes or PPH rates compared to an actively managed third stage of labour. Although longer third stages were more likely with physiological management, no significant differences were observed. Retained placenta levels and blood transfusion requirements were low. Hemoglobin measurement showed no significant differences. Increased uterotonic treatment was required in the physiological group
Begley, 2012 Irish and New Zealand	To explore the views and the skills Irish and New Zealand midwives employ in expectant management of the third stage of labour (EMTSL)	Qualitative descriptive study, semi structured one‐to‐one interview	The carer anticipates natural uterine contractions and oxytocin rise at birth to separate and expel the placenta, without administering a prophylactic drug or using cord‐clamping or controlled cord traction	Routine administration of a prophylactic uterotonic.	27 midwives who used EMTSL at least 30% of the time, and who averaged PPH rates of less than 4% in those births	Four themes were identified: ‘Going with the flow’, ‘Knowing it's separated,’ ‘Coping with the abnormal’ and ‘Letting it come.’ These elements of EMTSL add to midwifery knowledge and provide a basis for further discussion on how normal physiology can be supported during the third stage
Dixon, 2013; New Zealand	To describe, analyze and compare the midwifery care pathway and outcomes provided to a selected cohort of New Zealand women during the third stage of labour between the years 2004 and 2008	Retrospective cohort	No prophylactic drug is given, controlled cord traction is not used, clamping and cutting of cord is delayed, only maternal effort to expel placenta after signs of separation	Uterotonic given (prophylaxis) following birth of baby or with anterior shoulder at birth, cord is cut and clamped, controlled cord traction after signs of separation		The rates of physiological third stage care (expectant) and active management within the cohort were similar (48.1% vs. 51.9%). Women who had active management had a higher risk of a blood loss of more than 500 mL, the risk was 2.761 when a woman was actively managed (95% CI: 2.441–3.122) when compared to physiological management. Women giving birth at home and in a primary unit were more likely to have physiological management
Saxton, 2016	A psycho biologically based midwifery theory aimed at describing, explaining and predicting how to minimize the risk of PPH for individual women, and the rate of PPH for populations of childbearing women	Theory description	Pronurturance care (skin to skin and breastfeeding within 30 min after birth)	NA	NA	Pronurturance Plus theory is designed to describe, explain and predict midwifery activity in the third and fourth stages of labour to create eutony and eulochia. Pronurturance promotes the woman's focussed attention and parasympathetic dominance which, together optimizes her reproductive psychophysiology facilitating eutony and eulochia
Erickson, 2019 USA	To examine risk for total postpartum blood loss, PPH, and blood transfusion, and evaluate outcomes associated with active management of the third stage for women undergoing physiologic birth	Latent class approach	No AMTSL, No modified AMTSL	AMTSL: prophylactic oxytocin, controlled cord traction, and uterine massage after placental delivery. “Modified AMTSL: oxytocin being given after the placenta was delivered, or no umbilical cord traction for expediting third stage	2322 vaginal births were included in the generalized linear modeling and 15 physiologic birth and covariate factors met criteria to be considered in latent class analysis	The study categorized women into four classes: physiologic (44%), dysfunctional (35%), preterm (44%), and multiparous (40%). The use of AMTSL for women with a physiologic birth was associated with higher risk for preterm pregnancies and prolonged third stage labor
Baker, 2020 Study two, UK	Study two: to explore the practicability of midwives conducting active and expectant management approaches in the midwife‐led units	Multi‐method research Study Two: qualitative approach, using semi structured interviews	Support the woman during labor and birth by providing a warm, calm environment, encouraging skin‐to‐skin contact, and waiting for signs of placental separation. Breastfeeding or nipple stimulation can also stimulate the physiological release of oxytocin	A prophylactic uterotonic drug (exogenous oxytocin) is given, together with delayed cord clamping and cutting of the cord and controlled cord traction after signs of separation of the placenta	6 midwives were interviewed, using semi structured interviews	4 themes emerged: “The woman’; ‘The Midwife’; ‘Working within an organization’; and ‘Recent changes in childbirth’ These themes suggested that the midwives interviewed see their third stage of labour practice as shaped by varied and sometimes contradictory considerations that influence their interactions with the women they cared for, the midwives themselves, their colleagues and also the wider organizational and ideological context
Backer, 2021	To review evidence on the effectiveness of active or expectant third stage management approaches for low‐risk women in midwife‐led units or home settings seeking minimal intervention	Narrative critical literature review	Midwives monitor placental separation, ensuring spontaneous or gravity‐assisted delivery and protect oxytocin release by the woman's body	A prophylactic uterotonic drug is given, in association with delayed cord clamping, cutting of the cord and controlled cord traction	n.a.	Research suggests expectant management risks may be due to practitioner's limited training, confidence, or experience, rather than the management approach. It may be more suitable for low‐risk women in midwife‐led units or home settings
Baker, 2022a, Northwest England	To examine the relationship between third stage of labour management approaches, and incidence of postpartum hemorrhage and severe postpartum hemorrhage in women birthing in midwife‐led units	Retrospective cohort	No routine use of uterotonic drugs, no clamping of the cord until pulsation has stopped and delivery of the placenta by maternal effort	Syntometrine is administered before cord clamping and cutting, usually within 5 min of birth, with controlled cord traction after placental separation	1268 women who had a normal vaginal birth at a midwife‐led units	Of the 765 women intending to receive active management and the 508 intending to receive expectant management, 9.54% and 14.0% experienced postpartum hemorrhage respectively (*p* = 0.015). Severe postpartum hemorrhage was experienced by 14 (1.83%) women intending to receive active management and 16 (3.66%) intending to receive expectant management (*p* = 0.134)
Backer, 2022b	To investigate the third stage management approaches, the incidence and treatment of postpartum hemorrhage in women giving birth solely in midwife‐led units	Systematic review	No routine use of uterotonic drug. No clamping of cord until cord has stopped pulsating and delivery of placenta by gravity or maternal effort	Use a prophylactic uterotonic drug, delayed or immediate cord clamping and controlled cord traction	9 identified studies	Active management has higher postpartum hemorrhage incidences compared to expectant management in all birth settings. However, twice as many women receiving expectant management receive further treatment for excessive blood loss compared to active management. Studies also show a lower incidence of postpartum hemorrhage in midwife‐led units despite increased expectant management and reduced active management

### 2.9 Data Analysis

Data analysis was informed by a basic qualitative content analysis [14] with the intent to identify broad patterns, similarities, and original insights. Starting from the research questions, which serve as guiding parameters for subsequent data extraction, investigators engaged in a comprehensive data extraction process. The development and application of a coding scheme form the cornerstone of the analysis, where researchers create and implement a structured framework to categorize extracted data systematically. This framework enables the identification of recurring patterns, themes, and relationships within the literature. The analysis culminates in a thorough synthesis of findings, where researchers integrate coded data to generate meaningful insights that address the initial research questions. The NVivo 15 software was employed.

## 3 Results

The search identified 2190 articles; after the removal of duplicates, 1779 records remained. Title and abstract screening resulted in 80 records identified for full text review. A further 63 records were removed at this point as they did not meet inclusion criteria, leaving 17 articles for the final review.

### 3.1 Characteristics of Studies

We found three systematic reviews [15, 16, 17], one narrative review [18], three retrospective cohort studies [6, 17, 19], one prospective cohort study [20], one latent class approach study [21], one cross‐sectional study [22], one exploratory study [23], two qualitative studies [24, 25], two doctoral theses [26, 27], and two theory description studies [28, 29]. Studies were mainly from the United Kingdom [15, 24, 26, 27] and New Zealand [6, 20, 24], with Fahy, Dixon, and Baker as the main researchers publishing on this topic. Most of the studies were conducted in multiple settings, including hospitals, midwifery units, and at home [6, 15, 20, 22, 27].

### 3.2 Key Findings

The results of the included studies offer a comprehensive description of expectant management during the third stage of labour, highlighting its complexity and nuances.

From the data analysis, four key findings emerged based on the review questions:

1. The definition of a theoretical framework for a physiological approach to the third stage of labour.
2. What constitutes midwifery care during the third stage of labour to keep it normal: The midwifery balancing act—trusting the woman's body while preventing postpartum hemorrhage.
3. What evidence supports a physiological approach to managing the third stage of labour?
4. Suggestion of new risk factors for postpartum hemorrhage.

#### 3.2.1 The Definition of a Theoretical Framework for a Physiological Approach to the Third Stage of Labour

Among the studies included in this scoping review, two theoretical frameworks were identified: the “Midwifery Guardianship Theory” [28] and the “Pronurturance Plus Theory” [29], providing a deep and broad definition of what defines a normal third stage of labour.

These two theories provide new insights into how to approach the third stage of labour physiologically. Hastie & Fahy's [28] theory aims to optimize a woman's reproductive psychophysiology by promoting and respecting her intrinsic power to birth. The theory suggests that labour continues until the placenta is born and the uterus is fully contracted. Distractions should be avoided not only during the first and second stages of labour but also during the third, ensuring a safe and warm environment. The woman should be actively engaged in the birth process, feeling empowered and focused on her baby, her body, and the experience of childbirth. Fear or hyper‐vigilance should be avoided, as they can be potential risk factors for postpartum hemorrhage (PPH). The midwife, acting as a guardian, maintains watchful and positive expectations for a natural physiological placental birth while promoting and supporting maternal–infant love. The mother should hold the baby skin‐to‐skin, and the midwife will observe if the mother remains mindful of her body and uterine contractions. The woman will observe her baby, respond to its movements, and recognize signs of being ready to breastfeed.

The “Pronurturance Plus Theory,” proposed by Saxton et al. in 2016, is a psycho‐biologically based midwifery theory aimed at optimizing the third stage of labour for all women, regardless of their risk of postpartum hemorrhage. This theory promotes focused attention and parasympathetic dominance, enabling “eutony” and “eulochia” [29]. Pronurturance involves immediate skin‐to‐skin contact and breastfeeding within 30 min of birth. Midwifery care during this phase should be focused on warmth, safety, and a peaceful environment. Warmth and skin‐to‐skin contact promote endogenous oxytocin release during the third stage, allowing parasympathetic dominance and the emergence of innate nurturing behaviors. Midwives should monitor both mother and baby unobtrusively, minimizing neocortical stimulation. The focus should be on mother and baby, with the baby's natural crawling toward the breast triggering further oxytocin release.

#### 3.2.2 What Constitutes Midwifery Care During the Third Stage of Labour to Keep It Normal

Ten of the included articles addressed how midwives provide care during the third stage of labour to keep it normal [6, 17, 20, 22, 23, 24, 25, 26, 28] and Harris [30]. These studies describe how midwives strike a balance between trusting the woman's body and ensuring safe care to prevent postpartum hemorrhage. This approach has been instrumental in shaping the understanding of midwifery care during this stage, described as follows:

“Trust the process and the woman's body” [24] is the first step to providing expectant management of the third stage of labour. Midwives are encouraged to carefully observe both the mother and baby during this time [17, 24, 25, 28] and recognize signs of well‐being.

Encouraging an upright position during the third stage has been widely advocated [17, 24]. Some midwives even suggest using the toilet as a practical method to help and support the physiological birth of the placenta [24].

Protect and guarantee prolonged skin‐to‐skin contact [17, 24, 28].

The placenta should be born only thanks to the mother's efforts [6, 17, 24]. Wait for signs of the placenta's separation and avoid touching the uterus [17]. However, studies suggest there is a debate among midwives on the opportunity to touch the uterus or not and how or when to do so, as described by Jangsten et al. [25]. Studies also suggest a debate on the opportunity to give prophylactic oxytocin to all women or only if necessary. This debate reflects two competing perspectives: adhering to policies and guidelines versus acting autonomously based on professional judgment [25]. For some midwives, it is tough to accept that someone else tells them what to do when they are the primary responsible party for the process [25].

Delayed cord clamping and cutting, and avoiding cord traction [6, 17, 24] unless a gentle ease is used to help the placenta lift out if it is clear it has been separated and is sitting inside the vagina. Begley et al. [24] described this as a “guilty secret.” In fact, the researcher describes how textbooks and guidelines tend to state that cord traction should be avoided when using expectant management of the third stage of labor, but midwives describe this technique as something different. It is not real cord traction but a gentle easing of the placenta, only used when they see the insertion of the cord or the bulging of the vagina [24].

Being prepared for complications, such as, PPH, is essential during this stage. Midwives are trained to carefully monitor the mother's progress, observe for signs of placental detachment, and engage in open communication with the woman about her sensations and experiences [25]. Moreover, this review reveals a wide range of ways midwives care for women during the third stage of labor, with at least nine different types of management presented [23]. Midwives often lack confidence in managing the third stage in a completely physiological way but tend to prefer a more expectant approach compared to physicians [22, 23]. Most midwives do not believe active management should be used with women at low risk of PPH, and they use an upright position to facilitate the birth of the placenta, nipple stimulation to release oxytocin, and skin‐to‐skin contact [22]. The studies included in the review highlight the difficulties midwives face in acting solely in accordance with guidelines and procedures. They suggest that when women are given information and empowerment to make informed decisions, a large proportion will choose the physiological third stage [22, 25].

#### 3.2.3 Is Expectant Management of the Third Stage Safe? An Open Debate

This review investigates the ongoing debate regarding the clinical effectiveness of physiological third‐stage care following a physiological labour and birth, focusing on low‐risk women and those giving birth in non‐hospital settings. This topic has been discussed since 2010, when Fahy reviewed existing RCTs comparing active and expectant management of the third stage of labour, particularly in terms of their applicability to low‐risk women for postpartum hemorrhage (PPH). Fahy [31] argued that the conclusions from existing trials should not be generalized to women at low risk.

In 2011, Dixon et al. conducted a new systematic literature review, demonstrating that expectant management of the third stage is both effective and safe for those with a physiological labour and birth if the woman is well and in good health. Later, Backer and Stephenson [19] conducted a new literature review, identifying inconsistencies in studies comparing active and expectant management, as well as variations in blood loss outcomes for women at low risk of hemorrhage, particularly those assessed as having normal physiological parameters in midwifery‐led units.

#### 3.2.4 New Risk Factors for Postpartum Hemorrhage

Postpartum hemorrhage (PPH) is a significant global concern with various risk factors identified in the medical literature. These include a previous history of PPH, abnormal uterine anatomy, overdistended uterus, placental anomalies, antepartum hemorrhage, hemoglobin levels below 10 mg per liter, coagulation disorders, obstetric or anesthetic interventions, intrapartum hemorrhage, uterine muscle exhaustion, intra‐amniotic infection, and drug‐induced uterine hypotonia. However, a physiological approach to the third stage requires a re‐evaluation of these risk factors. Research by Fahy [31] and Hastie & Fahy [28] identified additional risk factors for PPH based on midwifery theories, such as, an inappropriate birth environment, lack of midwifery guardianship, significant mental illness, drug use, lack of skin‐to‐skin contact, inappropriate stimulation of the prefrontal cortex, exhaustion, and disconnected maternal responses to the baby. These findings suggest that active management of the third stage should be recommended when these circumstances are present. Erickson et al. [21] also observed that active management of the third stage of labor may not be beneficial for women undergoing physiological birth, as those with higher rates of physiological birth experienced lower rates of PPH and blood transfusion.

## 4 Discussion

This scoping review highlights the challenges midwives face in balancing adherence to protocols and recommendations [22] with their trust in the body's natural birthing processes. The findings suggest that midwives must navigate a delicate balance between providing safe care to prevent postpartum hemorrhage and trusting the woman's physiological ability to give birth without intervention. This aligns with the philosophical underpinning of midwifery, which is woman‐centred, promotes physiological birth, and advocates for minimal intervention in normal childbirth [32]. The review identified a robust theoretical framework underpinning physiological third stage management, primarily through the “Midwifery Guardianship Theory” [28] and “Pronurturance Plus Theory” [29]. These frameworks emphasize creating an undisturbed environment that optimizes birthing physiology and mother‐infant bonding. This aligns with previous research highlighting the importance of minimizing interventions during physiological birth [33]. The findings demonstrate that midwives must balance safety considerations with trust in natural birthing processes, challenging the widespread adoption of active management protocols in hospital settings [22, 23].

Significantly, the review expands our understanding of risk factors for postpartum hemorrhage beyond traditional medical indicators. Environmental and psychological factors, including inappropriate birth environments and disconnected maternal responses, emerge as important considerations [17, 28]. This broadened perspective suggests the need for a more holistic approach to risk assessment and management during the third stage of labour.

Additionally, decisions regarding the management of the third stage of labour should be women‐driven [34]. However, in many hospital settings, active management seems to be the norm, even for low‐risk women. Despite this, most midwives do not believe that active management is necessary or mandatory for women at low risk of PPH [20]. Midwives, even those who may lack full confidence in managing the physiological third stage, often prefer an expectant approach overactive management, unlike physicians who are more inclined to intervene [22, 23].

## 5 Strengths and Limitations

For many women, a physiological birth of the placenta is an intrinsic element of a normal birth, and active management is considered an intervention to be used if necessary [7]. One of the main contributions of this review is the redefinition of what constitutes expectant management of placental birth. The findings of this review offer a deeper understanding of what expectant management means in terms of a different way of doing it. In particular, it can be a starting point for rethinking what usually happens in a labour room soon after the baby is born. For example, in terms of noises and distractions, protecting and guaranteeing prolonged and uninterrupted skin‐to‐skin contact, using an upright position for placental birth, and trusting the woman's body ability to birth the placenta.

According to these results, the third stage of labour should involve careful observation, monitoring physiological indicators, and being prepared to manage postpartum hemorrhage if necessary. This approach can be defined as “watchful attendance” [13]. Furthermore, the review provides new insights into the risk factors for postpartum hemorrhage. Beyond the well‐known medical risks, factors such as, an inappropriate birth environment, lack of midwife guardianship, serious mental illness, substance abuse, poor skin‐to‐skin contact, improper activation of the prefrontal brain, fatigue, and disjointed responses to the baby are identified as contributors to the risk of postpartum hemorrhage.

However, this review has certain limitations. Despite our best efforts to conduct an exhaustive literature review, we found it extremely difficult to bundle what constitutes midwifery care during the third stage. This was discussed widely among the researchers during the screening process. It is hoped that the definition derived from these data will serve as a foundation for a shared understanding of how to provide care during placental delivery. Additionally, our review focused on qualitative and quantitative studies, literature reviews, and unpublished works. In future studies, we suggest including position statements, guidelines, and policy documents to further enrich the understanding of midwifery care in this context.

This work aims to be a starting point for future research. Based on our findings, we strongly believe that future studies should prioritize investigating midwifery care practices that support the physiology of the third stage and the efficacy of expectant management in physiological birth. It would be particularly beneficial to explore these practices in different settings, such as, home births or midwifery‐led units. Ultimately, these findings can help guide care providers in discussing options with women and improving placental birth practices.

## 6 Conclusion

Midwifery care during the physiological third stage of labour is crucial for women, even during placental birth. The increasing introduction of active management of the third stage of labour has raised concerns about the safety of physiological management of the third stage of labour following a physiological birth. It is essential for midwives to balance safety, preventing postpartum hemorrhage, while trusting the woman's body to complete the process without intervention. Midwives' decision‐making regarding the third stage of labour should be women‐driven, aligning with a woman‐centred approach to care. However, in hospital settings, active management remains the norm even for low‐risk women, despite evidence suggesting that many midwives prefer a more expectant approach. This paradox highlights the need for more flexible hospital guidelines that recognize both women's preferences and the safety of physiological management.

A specific theoretical framework underpinning this care emphasizes the importance of a natural, undisturbed third stage to maximize birthing physiology and mother‐infant bonding. Consequently, the midwife's role during this stage should involve creating a safe and calm environment, free of distractions, allowing the woman to focus on her baby, body, and the birthing process. Midwives should encourage mother‐infant bonding while maintaining a vigilant but optimistic approach toward spontaneous placental birth. Based on these findings, further research is essential to explore the effectiveness of expectant management of the third stage in various birth settings, such as, home births or midwifery‐led units. Midwifery practice should evolve to meet the individual needs of women, especially in low‐risk cases, while ensuring appropriate monitoring to prevent complications. Future studies should also focus on the long‐term impact of a physiological approach, not only on maternal outcomes but also on neonatal well‐being, particularly in terms of bonding and clinical results. Additionally, greater awareness of the environmental and psychological conditions influencing the third stage could significantly improve the quality of care provided, promoting a midwifery practice grounded in physiology and respectful of women's individual choices.

## Conflicts of Interest

The authors declare no conflicts of interest.

## Data Availability

The data that support the findings of this study are available from the corresponding author upon reasonable request.
